# Strengthening Ethiopia's Pandemic Preparedness and Response: The Role of Higher Education Institutions

**DOI:** 10.4314/ejhs.v35i3.1

**Published:** 2025-05

**Authors:** Eyob Girma Abera, Esayas Kebede Gudina, Temesgen Kabeta Chala, Daniel Yilma

**Affiliations:** 1 Department of Public Health, Jimma University, Jimma, Oromia, Ethiopia; 2 Clinical Trial Unit, Jimma University, Oromia, Ethiopia; 3 Department of Internal Medicine, Jimma University, Jimma, Oromia, Ethiopia; 4 Department of Health Policy and Management, Jimma University, Jimma, Oromia, Ethiopia

In the beginning of the 21st century, the world has faced unprecedented challenges, becoming more intense and less predictable (1). A multitude of stressors and shocks, caused by climate change, political instability, infectious diseases, and economic volatility, have become more common and pose a significant threat to humanity. The recent global COVID-19 crisis has brought about both a public health catastrophe and a profound economic crisis, amplifying existing dynamics of inequality as well as state fragility. This presents a critical challenge to governments and multilateral institutions to develop and implement an effective and cohesive response.

The causes of these extreme and unexpected events or disasters are complex and multifactorial. Their distribution also follows a stochastic nature requiring a new perspectives and robust methodologies to avert their unintended consequences over time (2). One of the strategies may include developing and establishing important metrics to monitor progress towards improving capacity to respond to public health emergencies.

Ethiopia's comprehensive emergency preparedness strategy, led by the Ethiopian Public Health Institute (EPHI), aims to establish a network of Public Health Emergency Operation Centers (PHEOCs) at both national and subnational levels. These centers align with global standards, particularly the International Health Regulations (IHR 2005), and are intended to serve as command centers during public health emergencies. Their main responsibilities include conducting risk assessments, tracking cases, coordinating laboratory work, managing emergency logistics, and information dissemination (3). However, for these centers to function optimally and sustainably, they must be supported by ongoing capacity-building, digital integration, and long-term financing mechanisms.

One of the most effective early measures in Ethiopia against the COVID-19 pandemic was the rapid establishment and activation PHEOCs. The national PHEOC was operational as early as January 27, 2020, nearly two months before the country confirmed its first case. This demonstrated proactive leadership and coordination under the Incident Management System (IMS) led by the EPHI (4). Most of the subnational Emergency Operation Centers were established and activated in higher education institutions (HEIs) through mobilizing HEI human power and infrastructures.

The response of HEIs during the COVID-19 pandemic and in other infectious disease outbreaks (5) has highlighted significant untapped local potential and capabilities. HEIs can play a crucial role in disaster risk reduction through multiple avenues: conducting research to identify and assess risks and impacts, formulating strategies for prevention, mitigation, and response, and designing and implementing training programs in disaster risk management. Furthermore, HEIs are well-positioned to generate evidence that can inform public discourse and influence policy in support of disaster risk reduction and management.

To address future potential pandemics and the recurring emergencies that communities face, there is an urgent need for sustained engagement of key stakeholders, particularly HEIs, in public health emergency management (PHEM). HEIs should consider integrating PHEM into their institutional mandates and academic curricula, including playing a key role in the development of strategic plans to detect, prevent, and respond to public health emergencies within their campus communities and surrounding regions. Investing in human resources, infrastructure, supply chains, and information systems at the regional level will ensure that emergency response is both rapid and equitable (6).

Local ownership and investment are key to ensuring the sustainability and effectiveness of PHEOCs. Ethiopia's Health Sector Transformation Plan II (HSTP-II) identifies epidemic preparedness and response as strategic priorities for building a resilient health system. Therefore, it is crucial to formalize the role of HEIs in public health emergency preparedness and response and ensure their sustainable engagement in PHEM through a comprehensive national strategy and allocation of adequate resources.
